# Tau protein plays a role in the mechanism of cognitive disorders induced by anesthetic drugs

**DOI:** 10.3389/fnins.2023.1145318

**Published:** 2023-03-01

**Authors:** Zheping Chen, Shenghan Wang, Zhaoqian Meng, Yuyang Ye, Guoliang Shan, Xinyue Wang, Xin Zhao, Yanwu Jin

**Affiliations:** Department of Anesthesiology, The Second Hospital, Cheeloo College of Medicine, Shandong University, Jinan, China

**Keywords:** tau protein, phosphorylation, anesthetic drugs, cognitive impairment, sevoflurane

## Abstract

Cognitive disorders are mental health disorders that can affect cognitive ability. Surgery and anesthesia have been proposed to increase the incidence of cognitive dysfunction, including declines in memory, learning, attention and executive function. Tau protein is a microtubule-associated protein located in the axons of neurons and is important for microtubule assembly and stability; its biological function is mainly regulated by phosphorylation. Phosphorylated tau protein has been associated with cognitive dysfunction mediated by disrupting the stability of the microtubule structure. There is an increasing consensus that anesthetic drugs can cause cognitive impairment. Herein, we reviewed the latest literature and compared the relationship between tau protein and cognitive impairment caused by different anesthetics. Our results substantiated that tau protein phosphorylation is essential in cognitive dysfunction caused by anesthetic drugs, and the possible mechanism can be summarized as “anesthetic drugs-kinase/phosphatase-p-Tau-cognitive impairment”.

## 1. Introduction

Cognitive disorders are mental health disorders that can affect cognitive abilities, including learning, perception and memory. Postoperative delirium (POD) refers to an acute cognitive dysfunction that occurs during the perioperative period, most of which are transient attacks but may also occur repeatedly. Its incidence rate increases with an increase in patient age. Postoperative cognitive dysfunction (POCD) has been reported as another symptom that lasts longer (Daiello et al., 2019). POCD is a sequela of the postoperative nervous system, which is related to the occurrence and persistence of central inflammation. It is characterized by excessive activation of glial cells in the brain, accumulation of abnormally phosphorylated tau protein (p-Tau) in the brain, and tangle and apoptosis of neurons (Wang et al., 2017; Chen and Mobley, 2019). The incidence of short and long-term declines in memory, learning, attention, and executive functions after anesthesia and surgery is reportedly high (Moller et al., 1998; Lewis et al., 2004; Hanning, 2005). An increasing body of evidence suggests that POCD is an age-related complication with high prevalence in elderly surgical patients (Monk et al., 2008; Ramaiah and Lam, 2009). POCD can significantly increase the risk of postoperative complications, prolong hospitalization and increase mortality (Steinmetz et al., 2009).

Emerging evidence suggests that neuroinflammation plays an important role in the pathogenesis of POD and POCD, mainly related to neuroinflammation caused by oxidative stress of brain tissue, blood-brain barrier damage and surgical trauma (Ren et al., 2014; Frank et al., 2017). Neuroinflammation can lead to excessive microglia and astrocytes activity, neurogenesis damage, synapse damage, neuronal dysfunction and death (Alam et al., 2018). Current evidence suggests systemic inflammatory reaction can occur as early as 4 hours after laparotomy (Kalff et al., 2003).

Surgery and anesthesia can cause POCD, which has been associated with cardiac surgery (Monk et al., 2008), non-cardiac surgery (Lee et al., 2005; Silverstein et al., 2007) and minor surgeries (Canet et al., 2003). It has been reported that about one-third of patients over 65 develop POCD after surgery, and 70% of them will develop dementia 3–5 years later (Vanderweyde et al., 2010) (Moller et al., 1998). Using anesthetic drugs can also affect the occurrence of POCD in different ways (Liu L. et al., 2022).

Tau protein is a microtubule-related protein mainly located in the axons of neurons important for microtubule assembly and stability, and its biological function is mainly regulated by phosphorylation (Iqbal et al., 2005; Polydoro et al., 2009; Buée et al., 2010). Therefore, the abnormal phosphorylation of tau protein can lead to its abnormal function and then affect the function of neurons. Some studies have shown that the hyperphosphorylation of tau protein triggers the degeneration or apoptosis of nerve cells and may lead to cognitive function damage (Lewis et al., 2000; Tanemura et al., 2002; Santacruz et al., 2005). The accumulation of p-Tau can reportedly disrupt the stability of the microtubule structure, leading to the destruction of the synaptic structure of neurons in the brain and the interruption of material and signal transmission until the death of neuron cells, which is considered to be the main pathological feature of neurodegenerative diseases such as Alzheimer’s disease (AD) (Congdon and Sigurdsson, 2018). It has been found that tau protein aggregate into neurofibrillary tangles (NFTs) after hyperphosphorylation. However, the formation of NFTs is widely thought as the main mechanism of tau protein hyperphosphorylation, leading to neurodegeneration and cognitive impairment (Grundke-Iqbal et al., 1986; Iwakiri et al., 2009). Recent studies have found that p-Tau accumulation before NFT formation can cause cognitive impairment (Berger et al., 2007; Kimura et al., 2007). At present, there is a rich literature available suggesting that p-Tau can lead to its abnormal deposition in neurons, which is the first step of the cognitive dysfunction cascade reaction (Wan et al., 2007; Kátai et al., 2016). However, many basic studies and some clinical studies have found that anesthetic drugs can cause significant phosphorylation of tau protein, accompanied by cognitive impairment (Weingarten et al., 1975; Planel et al., 2007; Run et al., 2009; Tan et al., 2010; Tang et al., 2011; Le Freche et al., 2012). Overall, this paper provides a comprehensive overview of the mechanism underlying cognitive dysfunction induced by perioperative anesthetic drugs mediated by tau protein.

## 2. Tau protein

As early as 40 years ago, Weingarten first isolated a protein that plays a key role in microtubule assembly function in the porcine brain and named it tau protein (Weingarten et al., 1975). In 1986, Grundke-Iqbal used a monoclonal antibody of tau protein for immunohistochemical detection, and his experimental results showed that the main component of the paired helical filament (PHF) in the brain of AD patients was abnormally phosphorylated tau protein (Grundke-Iqbal et al., 1986). Subsequently, Wood used monoclonal antibodies against tau protein to immunolabel NFTs with light and electron microscopy (Wood et al., 1986). The results showed that the abnormal synthesis, modification or aggregation of tau protein eventually leads to the formation of tangles. These findings have attracted the attention of many researchers and paved the way for extensive research about tau protein over the following decades.

**FIGURE 1:**
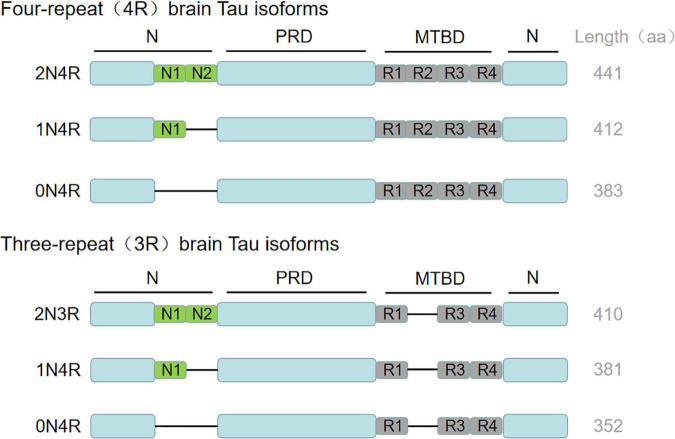
Tau biology: isotypic expression. *MAP*T gene produces six tau isoforms in human central nervous system through alternative splicing. The complete tau consists of N-terminal region, proline rich domain, microtubule binding domain (MTBD) and C-terminal region. The repetitive sequence constitutes the core of microtubule binding region and the core of PHF. The alternative splicing of exons 2 and 3 determines the number of N-terminal insertion sequences composed of 29 amino acids, which can form isomers (0N, 1N and 2N) with 0 or 1 or 2 N-terminal insertion sequences. Exon 10 was selectively spliced in MTBD to produce 3R and 4R tau. The C-terminal region is common to all subtypes. The amino acid length of each tau subtype is shown on the far right. aa, amino acid.

Tau protein is a microtubule-related protein encoded by the *MAPT* gene on chromosome 17q21 found in the frontal lobe, temporal lobe, hippocampus and other neurons in the brain (Goedert et al., 1989). Tau protein contains an N-terminal region, a proline-rich domain, a microtubule-binding domain (MTBD) and a C-terminal region, whereby the repetitive sequence constitutes the core of microtubule binding region and the core of PHF (Mandelkow et al., 2007). The *MAPT* gene contains 16 exons, of which exon 0 is a part of the promoter region, and exon 14 is a non-coding region. Both of them can be transcribed but not translated. Exons 1, 4, 5, 7, 9, 11, 12, and 13 are constitutive splicing exons, while exons 2, 3, and 10 can be selectively spliced. Besides, exons 4A, 6 and 8 are transcribed only in the peripheral nervous system. In the human central nervous system (CNS), six tau protein isomers are produced due to the selective splicing of exons 2, 3, and 10 (Kolarova et al., 2012; Stancu et al., 2019). The difference between the six tau protein isomers is that the number of repeats consisting of 31/32 amino acids in the MTBD is different. They are named R1, R2, R3, and R4 respectively. The splicing of exon 10 determines that the microtubule-binding domain of tau protein contains 3 or 4 repeats, leading to the formation of 4R tau or 3R tau. The alternative splicing of exons 2 and 3 determines the number of N-terminal insertion sequences composed of 29 amino acids, which can form isomers (0N, 1N and 2N) with 0 or 1 or 2 N-terminal insertion sequences (Muralidar et al., 2020; Uchihara, 2020). Therefore, according to the above differences, these six isomers of tau protein can be divided into 0N3R, 1N3R, 2N3R, 0N4R, 1N4R, and 2N4R. The putative mechanism is shown in Figure 1. Recent studies have found that the expression of tau protein is regulated by development. In the adult brain, six isomers of tau protein are expressed in CNS, while in the fetal brain, only the shortest tau isomer (0N3R) is expressed (Goedert et al., 1989). In addition, the tau protein is different in different species (Kosik et al., 1989) and brain regions (Boutajangout et al., 2004; McMillan et al., 2008).

Tau protein is widely expressed in the central and peripheral nervous system, the lungs, kidneys, the testis and other tissues. Tau protein is mainly distributed in the axons of neurons in CNS, participating in the assembly of microtubules and maintaining the stability of microtubules. The abnormal accumulation of tau protein is considered a neuropathological marker of AD. Tau protein dysfunction may lead to the collapse of the cytoskeleton, resulting in the dysfunction of nerve signal transmission and substance neurotransmitter transport. Disturbance of nerve signal transmission may lead to neurotrophic disorders. It has long been thought that tau protein is mainly concentrated in neuronal axons (Hirokawa et al., 1996). However, recent studies indicate that tau protein also exists in dendrites and may play an important physiological role (Ittner et al., 2010; Zempel et al., 2013).

### 2.1. Tau protein can be used as a biomarker of cognitive disorders

Tau protein and amyloid β-protein (Aβ) are hallmarks of AD and determine the occurrence and development of neurodegenerative diseases. Aβ is produced by the cleavage of amyloid precursor proteins in the brain. Among them, the two peptide forms composed of 40 and 42 aa residues are the most common, called Aβ-40 and Aβ-42 (Scarano et al., 2016). Recent studies have shown that, compared with Aβ, the aggregation of p-Tau is more closely related to the decline of early cognitive function and disease progression of AD (Spires-Jones and Hyman, 2014; Ossenkoppele et al., 2019), which may be an important starting factor for early memory decline, dementia and other symptoms and promote disease progression (Brier et al., 2016).

**FIGURE 2:**
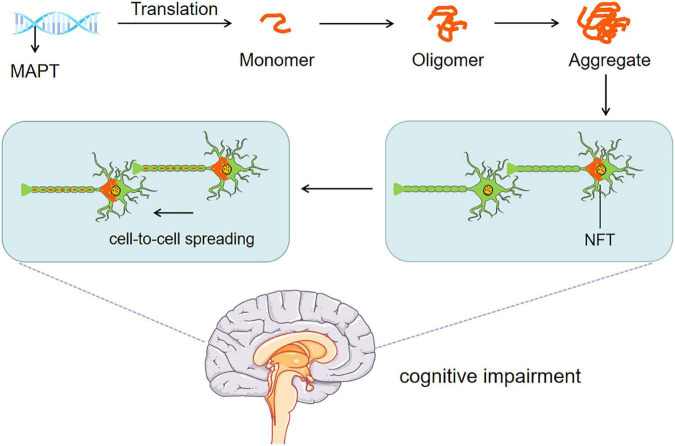
Tau pathomechanisms. The abnormal transcription and post-translational modification of *MAPT* gene intertwine with each other to form NFTs. NFTs affect axonal transport, synaptic plasticity, and cytoskeleton stability of neurons. Persistent diffusion and cell to cell p-Tau spreading eventually lead to cognitive impairment.

Threonine (Thr), serine (Ser) and tyrosine (Tyr) in tau protein are called phosphorylable sites. They are phosphorylated under the action of kinase to form p-Tau, which dissociates from the bound microtubule, leading to decreased microtubule stability and collapse of the cytoskeleton. The microtubule-associated protein tau is abnormally hyperphosphorylated and, in this altered state, is the major protein subunit of PHF that form NFTs, affecting the axonal transport of neurons, the plasticity of synapses, and the stability of the cytoskeleton, finally leading to dementia. Interestingly, phosphatase can remove the phosphate group on phosphorylated tau protein and restore the physiological function of tau protein (Souchet et al., 2022). The putative mechanism is shown in Figure 2. A meta-analysis showed that a large amount of tau protein was accumulated in the brain tissue of AD patients (Olsson et al., 2016). A recent study (Frontzkowski et al., 2022) found that young symptomatic AD patients showed stronger tau PET (Positron-Emission Tomography) accumulation in the frontal parietal lobe center (i.e., the area critical to maintaining AD cognition). Interestingly, the onset of AD symptoms was related to tau protein deposition, and the diffusion and deposition of tau protein were significantly related to cognitive dysfunction.

The clinical manifestations and pathogenesis of POCD and AD are similar, involving tau protein and Aβ plaques, central cholinergic injury, apolipoprotein E (ApoE) gene mutation, etc. It has been found that in patients undergoing hip replacement under subarachnoid anesthesia, IL-1β, Tau/Aβ1-42, pTau/Aβ1-42 and Aβ1-42 in the cerebrospinal fluid of POCD patients increased significantly, compared with the non-POCD group (Ji et al., 2013). A recent study (Liang et al., 2022) on 491 patients (65 years old or above) who received knee replacement, hip replacement or laminectomy found that preoperative plasma concentrations of phosphorylated tau at threonine 217 (p-Tau 217) and phosphorylated tau at threonine 181 (p-Tau 181) were related to postoperative delirium. A single-center case-control study showed that serum total Tau (T-Tau), p-Tau 217 and p-Tau 181 increased after major cardiac surgery. Only T-Tau was related to the incidence and severity of postoperative delirium, indicating a relationship between postoperative delirium and T-Tau. It was concluded that T-Tau could be used as a biomarker of postoperative delirium after major cardiac surgery (McKay et al., 2022).

### 2.2. Phosphorylation and dephosphorylation of tau

Protein phosphorylation refers to the addition of phosphate groups in three types of amino acid esters: serine, threonine, and tyrosine. Phosphorylation is the most common post-translational modification of tau protein. Tau protein phosphorylation level results from the balance between the active protein kinase and phosphatase (Martin et al., 2013; Wang et al., 2013).

It is well-established that p-Tau may induce pathological changes through four mechanisms. In this regard, p-Tau can cause synaptic damage by influencing intracellular tau location, changing tau degradation and truncation process, enhancing tau protein aggregation, and changing the relationship between tau protein and its interacting substances (Di et al., 2016; Wang and Mandelkow, 2016). More than 40 phosphorylation sites of Ser/Thr have been identified in the tau protein of AD, such as Thr181, Ser198, Ser199, Ser202, Thr205, Thr212, Ser214, Thr217, Thr231, Ser235, Ser262, Ser356, Ser396, Thr403, Ser404 and Ser422 (Wang et al., 2013; Kimura et al., 2018). Besides, these phosphorylation sites can be distributed in different domains of tau protein (Wang et al., 2013; Kimura et al., 2018). The interaction between tau protein and microtubule decreased significantly after phosphorylation at Ser262 and Ser356 sites. Quantitative studies *in vitro* showed that the microtubule-binding capacity of tau protein decreased by 35, 25, and 10%, respectively, after phosphorylation at Ser262, Thr231, and Ser235 sites. Therefore, the phosphorylation site of tau protein plays an important role in tau protein-induced neurodegeneration (Haase et al., 2004; Stokin and Goldstein, 2006; Majd et al., 2018). According to the latest research, an interdependent relationship hypothesis has been reported. The initial phosphorylation of one site regulates the phosphorylation state of another site. Accordingly, there are one/multiple phosphorylation master sites controlling tau phosphorylation at multiple sites. The four major sites, Thr50, Thr69, Thr181, Thr205 and two key kinases, GSK-3β and P38, are closely related to the regulation of p-Tau level (Stefanoska et al., 2022).

## 3. Ketamine

Ketamine is a pharmacological blocker of N-methyl-D-aspartate acid receptor (NMDAR), an ionic glutamate receptor subtype, which can bind with the tertiary structural unit of NMDAR encoded as 4TLM in protein data bank (PDB), occupy the ion channel site, and widely inhibit the normal physiological function of glutamatergic synapses (Johnson et al., 2015). Ketamine is a widely used anesthetic with hypnotic and analgesic properties.

Intriguingly, a study (Hudetz et al., 2009) showed that intravenous injection of 0.5mg/kg ketamine could reduce the occurrence of POCD 1 week after cardiac surgery, mediated by its neuroprotective role after cerebral ischemia through anti-excitotoxicity and anti-inflammatory mechanisms. In another study, 0.3 mg/kg ketamine was used for sedation and analgesia in patients undergoing ophthalmic surgery. After surgery, the Short Portable Mental Status Questionnaire was used to assess cognitive function. Consistently, it was found that ketamine could improve postoperative cognitive function (Rascón-Martínez et al., 2016). Although no positive findings have been documented in orthopedic surgery (Lee et al., 2015) and general anesthesia surgery (Mashour et al., 2018), ketamine can improve POCD in the short term after surgery according to the comprehensive meta-analysis results (Hovaguimian et al., 2018), and a meta-analysis showed that ketamine might reduce the incidence of POCD in patients with heart disease after surgery (Cui et al., 2020). The mechanism of ketamine in immediate improvement of POCD can be explained as follows: ketamine can be used as a neuroprotective agent both inside and outside cells: ketamine seems to reduce calcium-mediated cell death and excitotoxicity, reduce the release of proinflammatory cytokines, prevent micro thrombosis (through platelet inhibition and cerebral vasorelaxation) and promote neuronal growth (Li and Vlisides, 2016; Bell, 2017).

Current evidence suggests that long-term or continuous use of ketamine may significantly damage cognitive function. It has been reported that the language and memory ability of patients taking ketamine for a long time decreased to varying degrees (Ke et al., 2018). Kim et al. studied 30 patients who took ketamine for a long time to treat Complex Regional Pain Syndrome (CRPS). The results showed that compared with CRPS patients who did not receive frequent ketamine for a long time, their cognitive function was impaired (Kim et al., 2016). Another study (Liao et al., 2010) showed that the dosage dependence of white matter in the bilateral frontal lobe and left temporoparietal region was abnormal in patients with long-term use of ketamine, which may be the basis of microscopic changes in the brain after long-term use of ketamine. In addition, repeated use of ketamine can cause damage to multiple areas of frontal and medial temporal lobe function (Chan et al., 2013). Unlike its immediate effect, the low function of the glutamate system may be the mechanism underlying cognitive impairment related to chronic ketamine use, and inhibition of CaMKIIβ-ERK1/2-CREB/NF-κB signaling may mediate chronic ketamine use-associated cognitive impairments by restraining synaptic signaling (Luo et al., 2021). Growing evidence suggests that the brain changes caused by chronic ketamine treatment are long-term adaptive or plastic changes, not degenerative changes (Featherstone et al., 2012).

Importantly, Ketamine can significantly affect the phosphorylation process of tau protein. A study (Yeung et al., 2010) found that after 6 months of ketamine administration, the positive sites of hyperphosphorylated tau protein significantly increased in the prefrontal lobe and entorhinal cortex of mice and monkeys, and the long-term toxicity of ketamine may involve a neurodegenerative process similar to aging and/or Alzheimer’s disease. In another study (Li et al., 2019b), a long-term (6 months) ketamine administration model for wild-type (C57BL/6) and tau gene knockout mice was established to study the effects of different doses of ketamine (30 mg/kg, 60 mg/kg) on the expression and phosphorylation of tau protein in mice. The results showed that p-Tau was at Ser396 and Ser202/Thr205 (AT8) sites but not significantly increased at Ser199, Ser262 and Ser404 sites. Long-term administration of ketamine can reduce the hippocampal synaptic function and the number of membrane AMPA (α-amino-3-hydroxy-5-methyl-4-isoxazole-propionic acid) receptors by relying on the mechanism of tau protein phosphorylation at Ser202/Thr205 and Ser396 sites, revealing the role of tau protein phosphorylation in the mechanism of ketamine neurotoxicity. Subsequently, it was demonstrated that both single-dose and long-term use of ketamine could lead to the hyperphosphorylation of tau protein at Ser202/Thr205 and Ser396 (Li M. et al., 2020). A single dose of 80 mg/kg ketamine could increase GSK-3β activity and decrease PP2A activity. On the other hand, long-term administration of ketamine (60 mg/kg) increased GSK-3β and CDK5 activities and decreased PP2A activities. GSK-3β, CDK5 and PP2A may be related to ketamine-induced tau protein phosphorylation. A study reported that after intraperitoneal injection of 40 mg/kg ketamine in 7-day-old rats induced hyperphosphorylation of tau protein at the Ser404 site, leading to microtubule rupture and hippocampal neuron damage in the hippocampus of newborn rats (Jin et al., 2013). In addition, it has been found that 100mg/kg ketamine/methylthiazide induced tau’s rapid and powerful phosphorylation in a dose-dependent manner after 1 hour under normal and low-temperature conditions in mice (Hector et al., 2020). When ketamine and methylthiazide were combined, the phosphorylation sites included Ser396, Ser262 and other residues (such as Thr181 and Ser202/Thr205). In addition to tau protein phosphorylation, ketamine can affect the transport and secretion of tau protein. A recent study (Ren et al., 2022) found that 40 mg/kg ketamine could induce delirium-like behavior in mice and increase the content of serum tau in endosomes, which is mainly responsible for the transport of intracellular goods, including proteins, lipids and nucleic acids that are important for cell function, and key for neurons to release tau protein (Elkin et al., 2016). The multivesicular body (MVB) is a special subset of endosomes with intracavitary vesicles combined with membranes, and the intracavitary vesicles are essentially the precursors of exosomes. Ketamine can increase the number of endosomes, inhibit the maturation of endosomes, and increase the uptake of tau by endosomes, leading to the migration of endosomes to the cell membrane to form exosomes and release more tau protein from the cells.

**FIGURE 3:**
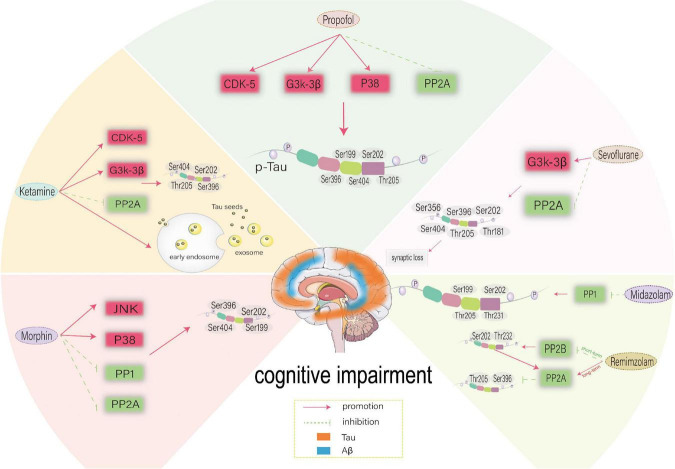
Possible mechanism of different anesthetic drugs-induced-Tau phosphorylation at different sites. CDK5, cyclin-dependent kinase 5; GSK-3β, glycogen synthase kinase-3β; PP2A, protein phosphatase 2A; PP2B, protein phosphatase 2B; PP1, protein phosphatase 1; CDK5, cyclin-dependent kinase 5; JNK, c-Jun NH2-terminal kinas.

Ketamine is one of the most commonly used drugs in clinical anesthesia for children and elderly patients and may affect cognitive function. The putative mechanism is shown in Figure 3. The results of the above clinical and basic experiments suggest that the impact of ketamine on human cognitive function may occur within a specific time window and that the dose and duration of ketamine treatment determine the immediate and long-term effect on cognitive function damage or protection. In addition, ketamine can play a protective role by reducing the neuritis caused by surgery (Zhao et al., 2020), which may account for the above differences.

## 4. Propofol

Propofol (2,6-diisopropylphenol) is an alkyl phenol derivative. Similar to other intravenous anesthetics (such as benzodiazepines and barbiturates), it can play a hypnotic role by activating the inhibitory neurotransmitter GABA (γ-aminobutyric acid) of the central nervous system (Brohan and Goudra, 2017).

Propofol is an intravenous anesthetic that has been widely studied for its effect on cognitive dysfunction. An international, prospective, randomized, multicenter, equivalence test of General Anesthesia compared to Spinal anesthesia (GAS) project compared the neurodevelopment of 2-year-old children under propofol general anesthesia and conscious local anesthesia in treating indirect inguinal hernia. The results showed that if the duration of general anesthesia is less than 1h, the behavioral/developmental assessment of children showed that general anesthesia did not affect their neurocognitive development (Chinn et al., 2016). A two-way study, Pediatric Anesthesia Neuro Development Assessment (PANDA), compared the neurodevelopment of patients who received hernia repair under general anesthesia before age 3 with patients in the same level control group. The average time of exposure to general anesthesia in children was about 80 minutes. The neuropsychological study showed no difference in the neural memory function between the two groups. In both experiments, no significant decrease in human cognitive ability was observed due to short-term propofol anesthesia exposure (Chinn et al., 2016). A meta-analysis showed that at 24 h, 3 days and 7 days after hip surgery, propofol could cause minimal changes in cognitive function (Bhushan et al., 2022). However, when using single anesthetic propofol for cystoscopy or hysteroscopy, the incidence of cognitive dysfunction in elderly patients (≥73 years old) after surgery on the first day was 47% (Rohan et al., 2005). Interestingly, it has been found that propofol can damage children’s short-term memory after surgery and recovery to the baseline level 3 months after surgery, with limited impact on long-term cognitive function (Yin et al., 2014). The long-term effects of propofol on cognitive function warrant further study. Although propofol anesthesia may cause a certain degree of cognitive impairment in the short term, it is far less than inhalation anesthesia (Hou and Xiao, 2019; Negrini et al., 2022). A meta-analysis comparing the effects of propofol and sevoflurane anesthesia on the postoperative cognitive function of elderly patients with lung cancer (>60 years old) suggested that propofol yields more adverse effects on the cognitive function of elderly patients with lung cancer than sevoflurane (Sun et al., 2019).

A study (Whittington et al., 2011) reported that 30 min after administration of 250 mg/kg propofol intraperitoneally, tau phosphorylation was significantly increased on AT8 (Ser202/Thr205), CP13 and PHF-1 phosphorylation in hippocampus and AT8 (Ser202/Thr205), PHF-1, MC6, pS262 and pS422 epitopes in the cortex. In both brain regions, tau phosphorylation persisted at the AT8 epitope 2 h after propofol administration, and the level of tau protein phosphorylated at AT8 returned to the control level 6 hours later, which may be attributed to the fact that PP2A inhibition is responsible for the hyperphosphorylation of tau at multiple sites 30 minutes after propofol exposure. At normal body temperature, propofol can also directly affect tau phosphorylation. However, previous studies have shown that the mechanism of inhibiting PP2A activity induced by hypothermia after intravenous injection and inhalation of anesthetics induces tau phosphorylation (Planel et al., 2007). A study (Huang et al., 2016) further found that continuous infusion of propofol for 2 h under normal and low-temperature conditions can increase p-Tau, while temperature control can reduce p-Tau to a certain extent. Propofol significantly increased tau phosphorylation of AT8, AT180, Thr205 and Ser199 in the cortex and hippocampus except for Ser396 at low temperatures. Under similar temperatures, propofol can still cause a significant increase of AT8, Thr205 and Ser199 in the cortex and hippocampus. The specific mechanism may be that propofol induces hyperphosphorylation of tau protein in the hippocampus through GSK-3β and/or PP2A, and hypothermia can play a synergistic role through the same mechanism. It is widely thought that propofol-induced cognitive impairment in rats may be related to the hyperphosphorylation of tau protein leading to the re-entry of neurons into the cell cycle, which leads to apoptosis (Zheng et al., 2018). In addition, a study (Zhang et al., 2018) found that hypoxia combined with propofol could affect the expression of tau protein through the P38 mitogen-activated protein kinase (P38MAPK) pathway, thereby damaging the cognitive function of immature rats. The putative mechanism is shown in Figure 3.

Propofol is widely used for anesthesia during clinical practice. Previous studies confirmed that compared with inhalation anesthetics such as sevoflurane, propofol yields little damage to cognitive function, although short-term and long-term cognitive function damage remains controversial (Bilotta et al., 2014; Belrose and Noppens, 2019; Pang et al., 2021). Interestingly, it has been reported that in addition to the phosphorylation of tau, propofol impairs cognitive dysfunction, including the imbalance of calcium homeostasis in neurons (Yang et al., 2019), the release of proinflammatory factors (Popić et al., 2015), apoptosis (Fredriksson et al., 2007), and synaptic plasticity (Pearn et al., 2018). Nevertheless, propofol still has certain anti-inflammatory and neuroprotective properties (Shen et al., 2021; Tao et al., 2022). Therefore, an in-depth research is needed before consensus on propofol research is reached.

## 5. Benzodiazepine

In the 1950s, the first benzodiazepines with “sedation” characteristics, chlorodiazepines, were synthesized. Several derivatives have subsequently been produced, including many compounds used as drugs. GABA is an inhibitory transmitter of the central nervous system, and benzodiazepines bind to the extracellular part of the γ-aminobutyric acid type A (GABAA) receptor α and γ subunit interface, mainly regulating the activity of the inhibitory neurotransmitter GABA in the brain. At present, commonly used benzodiazepines include midazolam, diazepam, and the rapid-acting remimazolam (Kim and Fechner, 2022).

Clinically, the use of benzodiazepines is associated with an increased risk of short-term cognitive impairment (Hirshman et al., 2003; Chun, 2005). A prospective randomized controlled trial (Li et al., 2019a) on 164 elderly patients undergoing hip or knee replacement under combined spinal-epidural anesthesia (CSEA). Seven days after the operation, midazolam yielded a higher prevalence of POCD compared with dexmedetomidine. In another study, the Montreal Cognitive Assessment (MoCA) was used to assess the cognitive function of elderly patients over 65 from days 5–9 after elective major surgery. The results showed that benzodiazepines and isoflurane were independent predictors of POCD (Lertkovit et al., 2022). In addition to the short-term risk of cognitive impairment, it has been reported that benzodiazepines can increase the risk of cognitive impairment years or decades later. A prospective population-based study (Billioti de Gage et al., 2012) demonstrated that the risk of dementia increased by 50% within 15 years after using benzodiazepines. Another study (Billioti de Gage et al., 2014) studied the relationship between dementia risk and exposure to benzodiazepines over at least five years and found that benzodiazepines could significantly increase cognitive dysfunction.

Acute and chronic administration of benzodiazepines has been reported to lead to tau hyperphosphorylation *in vivo* (Whittington et al., 2019). The acute administration of 25 mg/kg midazolam in C57BL/6 mice was associated with a sustained significant increase in tau phosphorylation in the brain, including AT8 (Ser202/Thr205), CP13, AT180, and Ser199. The duration of pSer199 ranged from 30 min to 24 h. The specific mechanism may be the downregulation of PP1. 24h chronic infusion of midazolam 3mg/kg/h can result in p-Tau expression in the hippocampus for 1 week, including AT8 and CP13. The sustained sedative effect of midazolam may be the basis of cognitive impairment caused by long-term sedation in the ICU environment in clinical practice (Zhang et al., 2017; Spence et al., 2018). The putative mechanism is shown in Figure 3. A recent study (Liu X. et al., 2022) measured the memory function of aged mice immediately and one month after intraperitoneal injection of remimazolam. It was found that by reducing the expression of PP2B, a single intraperitoneal injection of remiazolam could increase p-Tau at Ser202 and Thr232 sites in the cortex of aged mice in a short period, thereby damaging the cognitive function of aged mice. However, long-term administration of remimazolam can reduce p-Tau of Ser396 and Thr205 by promoting the expression of PP2A. It is highly conceivable that p-Tau in the short term leads to long-term upregulation of PP2A, tau can provide feedback and activate the expression of PP2A, and the elevated level of PP2A delays tau phosphorylation and cognitive impairment in the process of natural aging. The putative mechanism is shown in Figure 3.

Both clinical and basic studies suggest benzodiazepines may aggravate short-term and long-term cognitive impairment. However, the short-term inhibition of the new benzodiazepine drug remimazolam on the cognitive function in elderly patients is reversible and can delay the decline of cognitive function in the long term, suggesting it may be a better choice for general anesthesia in the elderly (Liu X. et al., 2022).

## 6. Opioids

Opioid drugs refer to natural drugs and semi-synthetic derivatives derived from opium, which act on opioid receptors including μ, δ and κ (Rock and Parker, 2021). Opioids are the main drugs for induction of anesthesia, intraoperative maintenance and postoperative analgesia commonly used in clinical practice. It has the characteristics of quick onset and precise analgesic effect. However, due to the massive use or even abuse of opiates, many countries and regions have experienced an opioid crisis, which has caused serious harm to society (Chisholm-Burns et al., 2019; Baldo, 2021). The abuse of opioids in North America has led to the addiction of many people to opioids. In 2017, the number of deaths from excessive opioid use reached 60 million. The harm of opioids has aroused great concern around the world (Lirk and Rathmell, 2019). Initial exposure to opioids is often during surgery. Among the surgical patients receiving opioid treatment, 3–7% became chronic opioid users (Nobel et al., 2019). Addiction and drug dependence on opioids are potential risks associated with their use (Lalic et al., 2020).

Short-term use of opioids can increase the risk of POCD. A recent study (Awada et al., 2019) found that the incidence of cognitive impairment in patients with total knee replacement (TKA) in the 2-3 weeks after surgery is related to the excessive use of opioids after surgery. The acute use of morphine can lead to short-term anterograde and retrograde memory impairment (Friswell et al., 2008). The long-term use of opioids also has a significant impact on cognitive dysfunction. Another recent study (Neelamegam et al., 2021) analyzed the cognitive function of 2222 elderly patients aged 65–69 years using the Mini-Mental State Examination (MMSE) and found long-term use of opioids in the elderly can affect cognitive function. Non-medical prescription opioid use (NMPOU) is a major public health problem in the United States and Europe. Opioid users exhibit selective impairment during the assessment of cognitive empathy. The dose-dependent effect suggests that potential opioid-induced defects and the opioid system participate in the cognitive empathy process (Kroll et al., 2018). However, repeated long-term use of non-fatal opioids can lead to neurodegeneration similar to AD patients, resulting in a decline in cognitive ability (Voronkov et al., 2021).

The potential mechanism of cognitive dysfunction caused by the use of opioids is the phosphorylation of tau. People with high opioid usage have a significantly greater concentration of phospho-tau in the middle frontal gyrus than people with little-to-no opioid usage (Flanagan et al., 2018). Excessive AT8-positive NFTs have been found in opioid users. NFTs were found in the entorhinal cortex, subiculum, temporal neocortex, nucleus basalis of Meynert and the locus coeruleus (Ramage et al., 2005). Over-phosphorylated tau was deposited at Thr231 and Ser202 sites. Tau phosphorylated at Thr231 (AT180) was more common than Ser202 (AT8). Possible reasons for hyperphosphorylated tau deposition in drug abuse include hypoxic-ischemic injury, microglial cell-related cytokine release, and drug-related neurotoxicity or hepatitis. For drug addicts, hyperphosphorylated tau positive (AT8, AT100) nerve fibers were significantly increased in the frontal and temporal cortex and locus coeruleus, and GSK-3β increased, showing brain lesions related to early AD patients, leading to cognitive impairment (Anthony et al., 2010). In addition, the downregulation of CDK5 and P35 levels in the brain of opioid addicts can reportedly lead to the formation of NFTs (Ferrer-Alcón et al., 2003).

Morphine can induce tau phosphorylation at Ser199, Ser202, Ser396 and Ser404 sites in rat embryonic cortical neurons (Cao et al., 2013), which may be attributed to increased JNK and MAPKS that have been established to upregulate tau protein phosphorylation and amyloid protein deposition. P38 MAPK is related to morphine-induced neurotoxicity (Macey et al., 2006), and the phosphorylation level of P38 MAPK increases in a time-dependent manner after 10 μm morphine exposure. The activated P38 MAPK triggers JNK activation, which contributes to tau hyperphosphorylation and neurotoxicity in morphine-induced embryonic cortical neurons of rats (Cao et al., 2013). Tau phosphorylation at the AT8 site increased in the striatum, cortex and hippocampus following chronic opioid abuse (Kovacs et al., 2015). Repeated exposure to morphine increased the acoustic startle response and whole amygdalar levels of Aβ monomers and oligomers and tau phosphorylation at Ser396, but not neurofilament light chain levels. In addition, PP1, PP2A and PP3 can affect the phosphorylation of tau. Opioids can upregulate PP1-specific endogenous inhibitors, leading to increased p-Tau and aggravating the brain damage caused by repeated exposure to opioids (Liu et al., 2002). In addition, opioids can downregulate the activity of PP2A through demethylation, resulting in tau hyperphosphorylation (Chakrabarti et al., 2016; Park et al., 2018). The putative mechanism is shown in Figure 3.

The above research results suggest that opioids can affect cognitive function, and the perioperative abuse of opioids significantly increases this risk. An assessment of the abuse of postoperative opioid analgesics shows that only 28% of prescription drugs are taken, and only 5% of patients choose to return the remaining drugs to the drug enforcement administration-approved recycling sites. Most patients may retain opioids or discard them with garbage (Hill et al., 2017). Although opioids are often used as the basic drugs for perioperative analgesia, the long-term use of opioids after surgery is a major concern for clinicians. The study on tau protein shows that opioids can affect cognitive function through JNK/p38 MAPK participating in the phosphorylation of tau upstream. The current concept of multimodal analgesia and the extensive development of ultrasound-guided nerve block (Mayr et al., 2007; Elvir-Lazo and White, 2010; LaFontaine et al., 2022) may effectively reduce the use of opioids during the perioperative period and improve cognitive function.

## 7. Sevoflurane

On October 16, 1846, the first diethyl ether inhalation anesthesia operation was publicly displayed at the Massachusetts General Hospital, marking the birth of anesthesiology. Diethyl ether anesthesia is considered to be the beginning of modern anesthesiology. Over the following 110 years (1846–1956), diethyl ether has consistently been used in anesthetics. Sevoflurane is one of the most commonly used drugs in clinical anesthesia, especially for children. It has the advantages of fast induction, quick awakening, easy adjustment of the depth of anesthesia, light inhibition of the circulatory system, minimal respiratory irritation, etc. Accordingly, it is widely used in the induction and maintenance of pediatric anesthesia (Doi and Ikeda, 1993). The pharmacological basis of sevoflurane as an anesthetic is to cause amnesia, analgesia, coma and sedation by inhibiting the NMDA receptor (Petrenko et al., 2014). In addition, *in vivo* studies have suggested that GABAA receptors (Lim et al., 2014), voltage-gated sodium channels (Yokoyama et al., 2011) and nicotinic acetylcholine receptors (Tang et al., 2018) are potential targets for sevoflurane related to its hypnotic effects.

The existing research shows that sevoflurane can significantly affect cognitive dysfunction. Our previous study used MMSE and MoCA to measure the cognitive function of 90 patients aged 65–75 years indicated for esophageal cancer resection (Qiao et al., 2015). The results showed that the plasma concentrations of S-100β, TNF-α and IL-6 of patients under sevoflurane anesthesia increased compared with propofol. The incidence of POCD in elderly patients undergoing major surgery under sevoflurane inhalation anesthesia was higher than in elderly patients receiving intravenous propofol maintenance, while the incidence of POCD in elderly patients receiving methylprednisolone treatment was lower. Another study of 150 elderly patients that underwent laparoscopic cholecystectomy found that sevoflurane-induced anesthesia aggravated POCD compared with propofol (Geng et al., 2017). Under sevoflurane or propofol-based general anesthesia, there was no difference in the incidence of POCD 7 days after radical rectal resection, but sevoflurane yielded a more serious impact on cognitive function of elderly patients with mild cognitive impairment than propofol (Tang et al., 2014). A meta-analysis of the postoperative cognitive results of elderly patients undergoing non-cardiac surgery under intravenous anesthesia or inhalation anesthesia maintenance also proved that compared with intravenous anesthetics, inhalation anesthetics significantly increased the incidence of POCD (Miller et al., 2018).

In addition to the type of surgery, the patient’s age may also affect the incidence of POCD. The annual number of cases requiring surgery and anesthesia in infancy has been increasing recently. In the United States alone, about 6 million children (including 1.5 million newborns) receive surgery and anesthesia yearly (Servick, 2014). The results of a large number of animal experiments in the early stage showed that a variety of clinical anesthetic drugs have certain toxic effects on the developing nervous system (Slikker et al., 2007; Lasarzik et al., 2011), which suggests that early exposure of infants to anesthetic drugs may have an impact on their future intelligence and memory. Interestingly, a study (Fan et al., 2018) showed that prolonged sevoflurane inhalation anesthesia (≥3h) could increase the occurrence of POCD and was related to serum caspase-3, TNF-α, and IL-6 expression. Another recent study (Walkden et al., 2020) compared the results of neural development of 13,433 children and found that repeated exposure to sevoflurane was associated with an increased risk of poor motor function, low hand dexterity and low social scores in children. Although sevoflurane yields significant developmental neurotoxicity, it has been associated with a higher incidence of POCD in the elderly. The incidence of POCD in people over 60 has been reported to be at least twice that of the younger group (Alalawi and Yasmeen, 2018). With the gradual degeneration and aging of various organs, sevoflurane-based anesthesia in the elderly leads to a decrease in the lowest alveolar concentration of sevoflurane and an increase in the cumulative effect of sevoflurane (Cooter et al., 2020). Therefore, sevoflurane may last longer in the blood than in young people after anesthesia. Neuroinflammation in normal aging is a landmark change in brain aging (López-Otín et al., 2013). The specific mechanisms may involve the blood-brain barrier (BBB), A1-like astrocytes, micro activation and low-grade increase of inflammatory cytokines (Barrientos et al., 2015; Montagne et al., 2015; Cao et al., 2018). These age-related changes have led to the decline of age-related cognitive ability of the elderly (Rawji et al., 2016; Ben Haim and Rowitch, 2017). Sevoflurane exposure has been shown to lead to brain damage and cognitive dysfunction in aged rats, reducing the expression of C1q/tumor necrosis factor-related protein-3 (CTRP3) (Yang et al., 2020). In elderly animals, sevoflurane-related behavioral defects after anesthesia are often more severe and sustained than in young animals (Peng et al., 2020). In addition, sevoflurane can inhibit histone acetylation and cause cognitive dysfunction by enhancing the expression of acidic leucine-rich nuclear phosphoprotein-32A (ANP32A) in aging mice (Chai et al., 2022). Therefore, although sevoflurane is associated with considerable developmental neurotoxicity, it can increase the risk of cognitive impairment in elderly patients.

It has been established that a potential mechanism underlying sevoflurane-induced cognitive dysfunction is tau phosphorylation. Previous studies have shown that spatial learning and memory of hypothermic animals are impaired on the first day after inhalation of anesthetic anesthesia, and anesthesia and hypothermia lead to tau hyperphosphorylation at Thr205 and Ser396 sites in the hippocampus (Tan et al., 2010). A study (Le Freche et al., 2012) found that sevoflurane can cause phosphorylation of tau protein at room temperature in addition to inducing hypothermia. Acute sevoflurane anesthesia at room temperature resulted in dose-dependent and reversible tau phosphorylation in the hippocampus for 1 h at the end of the exposure. The specific sites were Ser396 and Ser404. In mice anesthetized with sevoflurane, tau phosphorylation on Thr181 was significantly increased, and repeated anesthesia led to persistent tau hyperphosphorylation and memory impairment. The underlying mechanism may involve the activation of Akt and Erk kinases, which lead to the inactivation of GSK3, an essential kinase in regulating memory and cognitive function (Peineau et al., 2007). Besides, Erk is also one of the key kinases in the memory process (Kelleher et al., 2004). Ser396 and Ser404 have also been documented in the literature as phosphorylation sites after sevoflurane anesthesia (Hu et al., 2013). A study (Tao et al., 2014) found that tau phosphorylation was caused by repeated exposure to sevoflurane at Ser202 and Thr205 rather than a single exposure to sevoflurane since sevoflurane specifically reduces the level of p-GSK-3β (Ser9), leading to the activation of GSK-3β. Sevoflurane induced tau phosphorylation at Ser202 and Thr205 residues in neurons, and tau was transmitted from neurons to microglia via extracellular vesicles (EV) or non-EV pathways, activating the NF-kB signaling pathway and leading to IL-6 generation and cognitive impairment (Dong et al., 2021). However, the relationship between tau protein and IL-6 seems to be much more than that. Recent research (Zhang et al., 2020) found that sevoflurane increases the phosphorylation of tau at Ser202 and Thr205, leading to the increase of IL-6 and inducing mitochondrial dysfunction in young mice. This interaction leads to synaptic loss and cognitive impairment in mice.

In addition, sevoflurane yields an age-dependent effect on cognitive function. Unlike in adult mice, the cascade of brain mitochondria-ATP-Nuak1-Tau has been documented in newborn rats, which may be a potential mechanism of age-dependent tau phosphorylation at Ser202, Ser356 and Thr205 and cognitive impairment after sevoflurane anesthesia in mice (Yu et al., 2020). In addition to age dependence, the phosphorylation of tau induced by sevoflurane may also be subject to gender differences. Compared with adult male mice, low brain testosterone concentration in newborn rats has been reported to lead to age-dependent tau phosphorylation and cognitive impairment after sevoflurane anesthesia (Yang et al., 2021). The underlying mechanism involves the activation of GSK-3β by sevoflurane mediated by reducing the phosphorylation level of GSK-3β Ser9. In addition to activating GSK-3β, it may also lead to enhanced interaction between GSK-3β and tau, leading to tau protein phosphorylation and long-term neuronal dysfunction. It is widely thought that testosterone may reduce tau phosphorylation at Ser202, Ser205 and Ser262 in young rats caused by repeated sevoflurane anesthesia. In addition to inhibiting the self-shedding and tangle of these phosphorylation sites, it can also inhibit the binding of tau protein with GSK-3β, making the phosphorylation of tau sites challenging, thus playing a protective role. Testosterone can regulate Aβ protein deposition and tau protein hyperphosphorylation in the hippocampus through androgen and estrogen pathways (Rosario et al., 2010). In addition, compared with other sex hormones, testosterone can effectively reduce mitochondrial function damage caused by Aβ protein and yield a protective effect on ATP production and mitochondrial membrane potential (Grimm et al., 2016). Compared with aging caused by age dependence, cognitive impairment may be more significant due to low testosterone levels caused by related diseases such as hypogonadism (Sherwin, 2003). In addition to GSK-3β, PP2A participates in the sevoflurane-induced phosphorylation of tau. Neonatal sevoflurane exposure leads to the reduction of PP2A activity and postsynaptic density protein 95 (PSD-95) levels, and sevoflurane can lead to synaptic damage of hippocampal neurons through the hyperphosphorylation of tau protein at AT8 (Ser202, Thr205), thus leading to cognitive dysfunction (Huang et al., 2022).

To sum up, sevoflurane can significantly affect postoperative cognitive dysfunction through tau phosphorylation. The putative mechanism is shown in Figure 3. In recent years, sevoflurane-mediated neuroinflammation (Tang et al., 2021), glucose amino acid metabolic disorder (Liu et al., 2015), calcium homeostasis disorder (Yang et al., 2008), autophagy and mitochondrial dysfunction (Xu et al., 2018), and synaptic plasticity (Li et al., 2019c) have also been extensively studied. These studies have consistently found that sevoflurane can affect cognitive function and neural development in different ways.

## 8. Dexmedetomidine

Dexmedetomidine is an imidazole compound, a dextral isomer of medetomidine, and a highly selective membrane binding G-protein coupling α_2_ adrenergic receptor agonist. In the 1980s, Segal discovered its sedation and hypnosis effects (Segal et al., 1988). In 1999, it was approved by the US Food and Drug Administration (FDA) for short-term sedation of mechanical ventilation patients in the adult intensive care unit (Gertler et al., 2001). Dexmedetomidine is widely used in clinical practice due to its sedation, analgesia, anti-anxiety, inhibition of sympathetic activity, mild respiratory inhibition, and stable hemodynamics (Bilotta and Pugliese, 2020).

A recent study (Wang et al., 2022) showed that dexmedetomidine reduced POCD and inflammation within 72 h after intubation in elderly patients. In another study, dexmedetomidine was intravenously injected 10 min before anesthesia induction to 119 patients with gynecologic cancer who had undergone laparoscopic extensive hysterectomy, with a load of 1, 0.5, and 0.25μg/kg at a rate of 0.2 μg/(kg⋅h) until 30 minutes before the end of the operation. 0.5 μg/kg dexmedetomidine during the perianesthetic period could effectively reduce the occurrence of adverse reactions and POCD and protect brain function (Huang et al., 2021). An increasing body of evidence from recently published meta-analyses suggests that, compared with other drugs (such as sevoflurane, propofol and benzodiazepines), dexmedetomidine can improve short-term and long-term cognitive function, especially in cardiac surgery (Li M. et al., 2020; Burry et al., 2021; Xiong et al., 2021; Yu et al., 2022).

Nonetheless, it should be borne in mind that dexmedetomidine is associated with a lower incidence of cognitive impairment compared with benzodiazepines such as midazolam (Riker et al., 2009; Burry et al., 2021). Moreover, compared with other sedatives, cognitive ability related to the clinical use of drugs has improved (Mirski et al., 2010), but dexmedetomidine can start tau hyperphosphorylation and aggregation. Dexmedetomidine can directly promote tau phosphorylation *in vivo* and *in vitro* (Huang et al., 2015). *In vitro*, only obvious tau phosphorylation was observed at the Ser396 site, and these changes disappeared after 6 h. However, dexmedetomidine hyperphosphorylated tau at AT8 and AT180 sites in the cortex and hippocampus under similar temperatures. Dexmedetomidine, similar to sevoflurane and propofol, can directly lead to the phosphorylation of tau protein at room temperature (Planel et al., 2007). Under normal temperature conditions, the acute administration of dexmedetomidine has been reported to lead to tau hyperphosphorylation in the hippocampus and cortex of mice (Whittington et al., 2015). Tau hyperphosphorylation in the hippocampus lasted 2 to 6 hours after dexmedetomidine administration, mediated by tau phosphorylation through the α_2_ adrenergic receptor (α_2_-AR). Dexmedetomidine is well-established to be an agonist of α_2_-AR; nonetheless, its role in tau protein phosphorylation and the specific signal pathways remain unknown. Although acute dexmedetomidine administration may phosphorylate tau protein through α_2_-AR, it was recently found that dexmedetomidine and clonidine, the agonists of α_2_-AR, can inhibit sevoflurane-induced tau protein phosphorylation and cognitive impairment by activating α_2_-AR (Sun et al., 2021).

To sum up, although dexmedetomidine can lead to tau hyperphosphorylation and aggregation, the interaction of various kinases of tau protein phosphorylation remains unknown, and dexmedetomidine does not cause cognitive impairment based on the literature on the use of dexmedetomidine which reported a low incidence of negative cognitive function. This phenomenon can be explained in the following ways: In addition to inducing tau phosphorylation, dexmedetomidine can induce apoptosis in rodents (Liu et al., 2016), which can reduce neuroinflammation and cognitive impairment in adult mice induced by high molecular group box 1 (HMGB1) and surgery (Hu et al., 2018). It can also prevent propofol-induced apoptosis and cognitive impairment in newborn rats (Wang et al., 2016; Shan et al., 2018).

## 9. Conclusion

Various anesthetics yield different effects on cognitive dysfunction by mediating tau protein changes (Table 1). Anesthesia has become an indispensable tool for current surgery since it can alleviate the physiological pain of patients undergoing surgery. However, the inhibitory effect of anesthetic drugs on brain function should be emphasized. The perioperative use of different anesthetics, the combination of different drugs, different concentrations of the same drug, or different administration methods in elderly surgical patients can lead to different degrees and incidences of cognitive dysfunction. The correlation between anesthetic drugs and cognitive dysfunction in perioperative patients has become a research hotspot in the study of cognitive dysfunction. In addition to elderly patients, many studies have shown that multiple anesthetic exposures are associated with an increased risk of learning disabilities and attention deficit/personality disorder in children (Sprung et al., 2012; Warner et al., 2018). Therefore, more emphasis should be placed on the selection of perioperative drugs.

**TABLE 1:** The effects of different anesthetic drugs on cognitive impairment mediated by Tau protein.

Study ID	Anesthetic drugs	Experimental subject	Dose	Outcome
Yeung et al. (2010)	Ketamine	Mice and monkey	1 mg/kg for 6 months for monkeys; 30 mg/kg for either 1, 3 or 6 months for Mice	p-Tau positive cells were found in the prefrontal and entorhinal cortices of mice and monkeys after ketamine administration for 3 months or above
Li et al. (2019b)	Ketamine	C57BL/6 mice and Tau knockout mice	30 and 60 mg/kg for 6 months	Long-term administration of ketamine can reduce the synaptic function of the hippocampus and the number of membrane AMPA receptors through the mechanism of tau protein phosphorylation at Ser202/Thr205 and Ser396 sites
Li M. et al. (2020)	Ketamine	C57BL/6 mice	20, 40, and 80 mg/kg for single administration; 30 and 60 mg/kg for 6 months for long-term administration	Long-term and single administration of ketamine increased the levels of p-Tau at Ser202/Thr205 and Ser396. A single administration of ketamine 80 mg/kg increased the activity of GSK-3β and decreased the activity of PP2A. Long-term administration of ketamine 60 mg/kg increased GSK3 β And CDK5 activity and decreased PP2A activity.
Jin et al. (2013)	Ketamine	7-day-old rats	40 mg/kg	Ketamine induced p-Tau at Ser404, leading to microtubule rupture and hippocampal neuron damage in the hippocampus of newborn rats
Hector et al. (2020)	Ketamine and xylazine	C57BL/6J mice	100 mg/kg for ketamine and 10 mg/kg for xylazine 140 mg/kg of ketamine with 14 mg/kg of xylazine	Ketamine/xylazine produced a marked c in a dose-dependent manner under normothermic conditions at Ser396, Ser262, Thr181 and Ser202/Thr205, and this phenomenon was exacerbated under hypothermic conditions
Ren et al. (2022)	Ketamine	C57BL/6J mice	40 mg/kg	Ketamine increased tau in the endosomes of cultured cells and the cell culture medium
Whittington et al. (2011)	Propofol	C57BL/6J mice	250 mg/kg	Propofol can increase p-Tau in the mouse hippocampus and cortex. In the hippocampus, p-Tau at the AT8 persists for at least 2 h following propofol administration before returning to control levels 6 h later.
Huang et al. (2016)	Propofol	SD rats	15 and 40 mg/h continuous infusion	Propofol with hypothermia significantly increased p-Tau at AT8, AT180, Thr205, and Ser199 in the cortex and hippocampus, except for Ser396. By maintaining the same temperature, propofol still induced significant elevation of AT8, Thr205, and Ser199 in the cortex and hippocampus.
Zheng et al. (2018)	Propofol	Wistar rats	30 mg/kg tail vein injection	Propofol-induced cognitive dysfunction in rats may be related to the p-Tau that causes neuronal cells to re-enter the cell cycle, thus leading to apoptosis
Zhang et al. (2018)	Propofol	SD rats	50 mg/kg	Under hypoxic conditions combined with propofol, p38 is activated, leading to increased p-Tau.
Whittington et al. (2019)	Midazolam	C57BL/6J mice	10 and 25 mg/kg; 3 mg/kg/h for 24 h	The acute administration of 25 mg/kg midazolam is related to the continuous significant increase of p-Tau in the brain at AT8 (Ser202/Thr205), CP13, AT180 and Ser199. Continuous infusion of midazolam 3 mg/kg/h for 24 h will result in p-Tau in the hippocampus lasting for 1 week at AT8 and CP13.
Liu X. et al. (2022)	Remimazolam	C57BL/6J mice	10, 12, 14, 16, and 20 mg/kg	Single intraperitoneal injection of remimazolam can increase p-Tau at Ser202 and Thr232 in the cortex of aged mice in a short time. However, long-term administration of remimazolam can reduce p-Tau at Ser396 and Thr205 by promoting the expression of PP2A.
Cao et al. (2013)	Morphine	SD rats	10 μM for 12 h	Morphine can induce p-Tau at Ser199, Ser202, Ser396 and Ser404. The specific mechanism may be increased JNK and MAPKS
Tan et al. (2010)	Isoflurane	SD rats	1.5% in 50% oxygen	p-Tau is not a direct result of anesthesia *per se*, but it is due to anesthesia-induced hypothermia leading to the inhibition of phosphatase activity as well as tau hyperphosphorylation
Le Freche et al. (2012)	Sevoflurane	C57BL/6J mice	acute (1 h) or repeated (five exposures of 1 h every month) anesthesia using 1.5 or 2.5% sevoflurane	Sevoflurane exposure is associated with increased p-Tau through the activation of both Akt and Erk pathways and spatial memory deficits
Hu et al. (2013)	Sevoflurane	7-day-old male rat	3% in 60% oxygen	Sevoflurane can induce p-Tau at Ser396/404.
Tao et al. (2014)	Sevoflurane	C57BL/6J mice and Tau knockout mice	3% in 60% oxygen	Sevoflurane can induce p-Tau at Ser202 and Thr205, GSK-3β activation, increase in interleukin-6 and reduction in postsynaptic density protein-95 levels in the hippocampus of young mice, and cognitive impairment in the mice
Dong et al. (2021)	Sevoflurane	C57BL/6J mice and Tau knockout mice	3% in 60% oxygen	Sevoflurane can induce p-Tau at Ser202 and Thr205, activate the NF-kB signaling pathway and lead to IL-6 generation and cognitive impairment
Zhang et al. (2020)	Sevoflurane	C57BL/6J mice and Tau knockout mice	3% in 60% oxygen 2 h daily for three consecutive days	Sevoflurane increased p-Tau at Ser202 and Thr205, and IL-6, induced mitochondrial dysfunction, synaptic loss and cognitive impairment
Yu et al. (2020)	Sevoflurane	C57BL/6J mice	3% in 60% oxygen 2 h daily for three consecutive days	A potential cascade of brain mitochondria-ATP-Nuak1-Tau exists in newborn rats, which may be the potential mechanism of age-dependent p-Tau and cognitive impairment after sevoflurane anesthesia
Yang et al. (2021)	Sevoflurane	C57BL/6J mice	3% in 40% oxygen 2 h daily for three consecutive days	Sevoflurane can enhance the interaction between GSK-3β and tau, leading to p-Tau and long-term neuronal dysfunction
Huang et al. (2022)	Sevoflurane	C57BL/6J mice	3% in 60% oxygen 2 h daily for three consecutive days	Sevoflurane exposure leads to a decrease in PP2A activity PSD-95 level. Sevoflurane can cause synaptic damage to hippocampal neurons through p-Tau at AT8 (Ser202, Thr205), thus leading to cognitive dysfunction.
Huang et al. (2015)	Dexmedetomi- dine	SD rat embryos	5, 10, or 20 μM	Dexmedetomidine increased p-Tau at the Ser396 epitope under hypothermic and normothermic conditions in the rat cortical neurons.
Whittington et al. (2015)	Dexmedetomi- dine	10-week old C57BL/6 male mice	30 and 300 μg/ml	Dexmedetomidine increases p-Tau and aggregation *in vivo* under normothermic conditions with the potential to impact spatial reference memory, and p-Tau is a dose-dependent phenomenon *in vitro*

**FIGURE 4:**
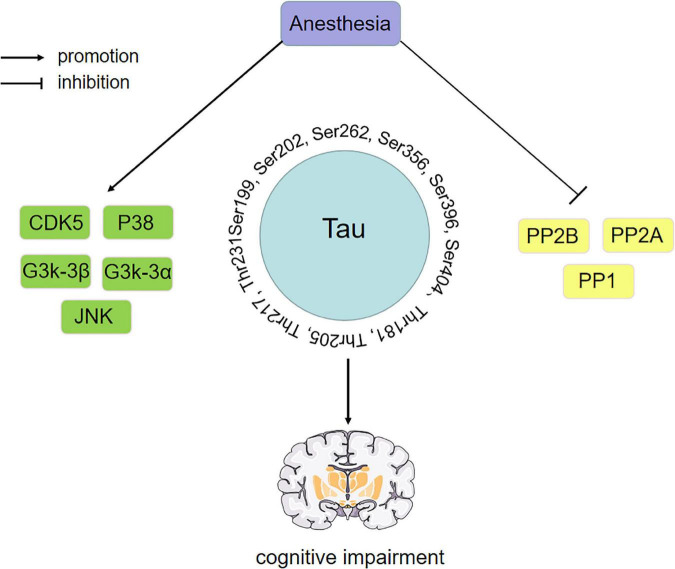
Anesthetic drugs-kinase/phosphatase-p-Tau-cognitive impairment. Key kinase/phosphatase includes GSK-3β, GSK-3α, CDK5, P38, JNK, PP1, PP2A, and PP2B. The phosphorylation sites of key tau include Ser199, Ser202, Ser262, Ser356, Ser396, Ser404, Thr181, Thr205, Thr217, and Thr231. The effect of anesthetic drugs on cognitive dysfunction through tau may be the result of the balance between various kinases and phosphatases. PP2A, protein phosphatase 2A; PP2B, protein phosphatase 2B; PP1, protein phosphatase 1; CDK5, cyclin-dependent kinase 5; GSK-3α, glycogen synthase kinase-3α; GSK-3β, glycogen synthase kinase-3β; JNK, c-Jun NH2-terminal kinas.

Cognitive function plays a determining role in our daily lives, which is significant for postoperative outcomes and long-term rehabilitation. Many studies have shown that anesthesia can cause tau phosphorylation, and tau is closely related to cognitive function. Therefore, studying how anesthetic drugs affect cognitive function through tau is essential. However, by comparing various experimental results, we found that anesthetic exposure showed significant differences in tau phosphorylation sites, which may be affected by different experimental environments, drug doses and administration speeds, treatment methods and time, and even individual differences. In addition, the phosphorylation level of tau protein is regulated by the expression or activity of related kinases and PP, although the levels and sites of kinases and PP affected by various drugs may differ. We found that some kinases and PP have been extensively studied during anesthesia exposure, which can be summarized as “anesthetic drugs- kinase/phosphatase- p-Tau -cognitive impairment”. The key kinases/phosphatases include GSK-3β, GSK-3α, CDK5, P38, JNK, PP1, PP2A and PP2B, while the key tau phosphorylation sites include Ser199, Ser202, Ser262, Ser356, Ser396, Ser404, Thr181, Thr205, Thr217 and Thr231. The influence of anesthetic drugs on cognitive dysfunction through tau may result from the balance between various kinases/phosphatases. The putative mechanism is shown in Figure 4.

Little is currently known about how to effectively reduce the harm caused by tau phosphorylation. Endocrine therapy represents a potential method, such as testosterone. The secretion of testosterone depends on the neuroendocrine mechanism of the hypothalamus-pituitary-gonad axis. Many clinical and basic studies have shown that testosterone yields a certain brain protective function, plays an important role in brain growth and development, and can inhibit the phosphorylation of tau to protect cognitive function (Rosario et al., 2010; Hall et al., 2015; Grimm et al., 2016; Mohamad et al., 2018; Yang et al., 2021). In addition, nanotechnology is widely thought to play a promising role in tau-targeted drug therapy. Nanocomposites can effectively penetrate the BBB to inhibit p-Tau, reduce oxidative stress, restore nerve damage and maintain neuronal morphology, thereby significantly improving AD mice’s learning and memory abilities (Zhou et al., 2020; Zorkina et al., 2020).

In addition to perioperative anesthetics, surgery is also a key element affecting cognitive dysfunction. Therefore, the potential relationship between anesthetic exposure, surgery and cognitive function has attracted significant interest. Anesthetic exposure can temporarily inhibit the relevant biomarkers of brain injury, triggering a temporary protective effect after acute injury in normal mice. However, in combination with surgery, relevant biomarkers have been reported to increase, and brain damage is aggravated (Dabrowski et al., 2012). Therefore, it was proposed that combining surgical trauma and anesthetic exposure would lead to a primary inflammatory reaction in the body, leading to neuroinflammation and synaptic damage (Fung et al., 2012). Neuroinflammation and synaptic damage are also important factors. Besides, AD or similar potential pathology may make individuals more vulnerable to the potential neurotoxic effects of surgical pressure and/or anesthetic exposure and increase the risk of postoperative cognitive impairment (Eikelenboom et al., 2011; Chang et al., 2021; Liang and Zhang, 2022).

To sum up, different anesthetics can affect perioperative patients’ short-term or long-term functional recovery through “anesthetic drugs-kinase/phosphatase-p-Tau-cognitive impairment”. However, the exact mechanism and the potential relationship between the stress caused by surgery and anesthetic exposure and cognitive function has not been fully understood. For anesthesiologists, it is essential to elucidate how to choose the type, dosage and administration mode of anesthetic drugs and avoid hypothermia induced by anesthetic drugs to reduce short-term and long-term cognitive dysfunction after surgery. In the short term, after surgery, the duration and dosage of some drugs, such as opioids, should be carefully considered. The development of anti-tau drugs may effectively reduce postoperative cognitive impairment. Over the past few years, many anti-tau drugs have been proven to effectively inhibit either reversing post-translation modifications that might drive the aggregation of tau, such as phosphorylation or directly preventing aggregation (Novak et al., 2017; Lu et al., 2018; van der Kant et al., 2019; Wang et al., 2021). In addition, to avoid using certain drugs, appropriate anesthesia methods, such as ultrasound-guided nerve block, CSEA and non-opioids anesthesia with spontaneous respiration, can be selected to effectively reduce the inevitable cognitive impairment caused by inhaled general anesthesia and intravenous anesthesia.

## Author contributions

ZC and YJ developed the idea. ZC reviewed the articles and wrote the manuscript. YJ and XZ revised and modified the manuscript. All authors approved the final version for publication.
